# Synthesis of Lipopeptides Using Vegetable Oils by Newly Isolated Strain of *Serratia marcescens* G8-1: Genomic Characterization and Process Performance

**DOI:** 10.3390/ijms26125794

**Published:** 2025-06-17

**Authors:** Slawomir Ciesielski, Wiktoria Stefańska, Kritika Singh, Ewelina Wielgus

**Affiliations:** 1 Department of Environmental Biotechnology, University of Warmia and Mazury in Olsztyn, Sloneczna 45G, 10-709 Olsztyn, Poland; 2 Department of Mechanical and Process Engineering, University of Applied Sciences Offenburg, Badstraße 24, 77652 Offenburg, Germany; 3 Centre of Molecular and Macromolecular Studies, Polish Academy of Sciences, Sienkiewicza 112, 90-363 Łódź, Poland

**Keywords:** biosurfactants, genomics, lipopeptides, *Serratia marcescens*

## Abstract

Biosurfactants are becoming increasingly popular, but industrial production of biosurfactants is difficult, partly due to high production costs resulting from the need to use expensive substrates. One economically feasible candidate is vegetable oils, which can be directly metabolized without pretreatment. The aim of this work is therefore to investigate the possibility of using vegetable oils for lipopeptide production by *Serratia marcescens* G8-1. The genetic background of this strain for the production of lipopeptides was investigated using a genomic approach. The biosurfactants were analysed by Ultra-Performance Liquid Chromatography coupled with Electrospray Ionisation Mass Spectrometry. The ability to reduce surface tension was investigated using a tensiometer. The results showed that the best effect in reducing surface tension was achieved by adding waste rapeseed oil. Sunflower and linseed oil also showed good results. Significantly poorer results were obtained when fresh rapeseed oil, sesame oil and pumpkin seed oil were used. The putative gene cluster for cyclic lipopeptides NRPS was identified in the genome of *S. marcescens* G8-1. The results obtained confirmed that serrawettin W1 is the major biosurfactant produced by *S. marcescens* G8-1. Of particular interest, the results showed the presence of vinylamycin when rapeseed oil was used.

## 1. Introduction

Biosurfactants are surface-active metabolites synthesized by various microorganisms. These substances are able to modify the hydrophobicity of the cell surface, which plays an important role in the adhesion of microorganisms to various surfaces [1,2]. Biosurfactants can reduce the surface and interfacial tension via the same mechanisms as chemical surfactants [3]. However, biosurfactants have many advantages over chemically synthesised ones, whether they are wholly or partially derived from materials of biological origin. Among the features that make biosurfactants commercial promising alternatives to chemically synthesized surfactants are their biodegradability, biocompatibility, low toxicity, wide applicability, and social acceptability [4,5]. Indeed, due to growing market demand for biobased products, biosurfactants are enjoying increasing interest. Most of work dealing with biosurfactants has focused on bioremediation and oil recovery, but more recently, a number of papers have focused on areas such as food and food-related industries, cosmetics, and biomedicine [3].

Biosurfactants are amphiphilic molecules with hydrophobic and hydrophilic constituents that allow them to create interfaces between fluids with different polarities. The hydrophilic portion can be composed of amino acids, anionic or cationic peptides, and carbohydrates. The hydrophobic tail usually contains peptides, proteins, or saturated or unsaturated fatty acids [6]. Biosurfactants are usually divided into high-molecular-mass molecules, which stabilize emulsions more effectively, and low-molecular-mass molecules, which reduce surface tension with greater efficiency [7]. The first group includes polysaccharides, lipopolysaccharides, proteins, and lipoproteins, whereas the second group includes glycolipids, phospholipids, and lipopeptides [8].

Among the low-molecular-weight biosurfactants, lipopeptides have been extensively investigated because of their excellent properties [9]. Microbial lipopeptides are amphiphilic molecules containing both polar and apolar moieties. The polar moiety is a cyclic peptide, while the apolar one is a linear or branched fatty acid that can be of various lengths and degrees of oxidation [10,11]. Cationic and anionic residues are available in the peptide portion, and sometimes non-proteinaceous amino acids are also present [12]. A wide range of lipopeptide structures have been identified, which display variation in the length and conformation of the lipid moiety, resulting in different homologues, while analogues exist due to variation in amino acid composition within the peptide moiety [13,14].

Because lipopeptide biosurfactants offer a great range of natural advantages for microbial cells, they can be synthesized by a wide range of bacteria. The most well-recognized lipopetides are produced by bacteria from the genera *Bacillus*, *Pseudomonas*, and *Paenibacillus* [15,16]. Less well-recognized are lipopeptides synthesized by *Serratia* species [14], which include serrawettin 1 (initially called serratomolide A), with identified homologous serratomolides B to G [14,17,18]. Additionally, other authors have identified serrawettin 2 [19] and serrawettin 3 [19]. *Serratia marcescens* is a widely known producer of serrawettin 1 and can also produce glycolipids, glucosoamine derivatives, and prodigiosin [14]. In *Serratia* cells, Serrawettin 1 is assembled by non-ribosomal peptide synthetases (NRPSs), similar to what occurs in other lipopeptide producers. NRPSs are large multi-modular enzymes employed in microbial secondary metabolism [20,21]. This system has been described at the molecular level [22] although knowledge of the genetic factors controlling the process of serrawettin synthesis is still incomplete.

Because of the extraordinary properties of serrawettins, such as their emulsification activity, surface activity, antitumor activity, antibacterial activity, and antifungal activity [14], these substances are desirable on the market. However, industrial production of biosurfactants is still limited, mainly due to their low profitability. Thus, different strategies to improve the economics of biosurfactant production are currently being tested. One of these strategies is examination of new strains that could synthesize lipopeptides more efficiently. A second strategy is the use of low-cost raw substrates, such as agro-industrial wastes; this could be a sustainable alternative to the use of more expensive substrates because the utilization of these residues contributes to the reduction of environmental pollution [23]. Along these lines, promising results have been obtained by Ferraz et al. [24], who tested the influence of vegetable oils on biosurfactant production by *S. marcescens*. These results suggest that examination of other plant oils could be beneficial for their utilization and economical production of lipopeptides at industrial scale.

Therefore, the main aim of this study was to test the potential of different plant oils for synthesis of serrawettin and other chemicals by a newly isolated strain of *Serratia marcescens* G8-1. As the most commonly cultivated oil plant in Poland is rapeseed, we also analysed waste oil obtained from this plant. Moreover, the genetic background of serrawettin synthesis was elucidated using a genomic approach.

## 2. Results

### 2.1. Surface Tension Analysis

*S. marcescens* G8-1 was cultivated in a medium with the addition of various plant oils in order to test their influence on serrawettin synthesis. Table 1 shows the surface tension values in the cell-free medium after 120, 144 and 168 h of cultivation. The decrease in surface tension in the sample without oil addition was between 29.84 ± 0.45 (120 h) and 31.28 ± 0.18 (144 h) mN/m, with applied critical micelle dilutions (CMD^−1^ and CMD^−2^) values of 48.41 ± 1.69 and 58.135 ± 2.22 mN/m, respectively. Of all the oils analysed, waste rapeseed oil gave the best results 40.38 ± 1.37 mN/m (mean value for three measurements). In this case, the values for CMD^−1^ and CMD^−2^ were 49.94 ± 1.13 and 52.16 ± 1.38 mN/m, respectively. The decrease in surface tension in the cell-free medium was similar when linseed oil and sunflower oil were added. In the case of linseed oil, the mean value for the measurement after 120, 144 and 168 h was 41.12 ± 0.64 mN/m with CMD^−1^ and CMD^−2^ values of 50.53 ± 2.05 and 55.1 ± 3.32 mN/m, respectively. The mean value for sunflower oil was 41.35 ± 0.91 mN/m with CMD^−1^ and CMD^−2^ values of 50.09 ± 2.32 and 53.11 ± 2.92 mN/m, respectively. A lower reduction in surface tension was observed when fresh rapeseed oil was added to the cultivation. In this case, the mean value for three measurements was 41.96 ± 1.26 mN/m with CMD^−1^ and CMD^−2^ values of 52.6 ± 3.33 and 56.12 ± 1.49 mN/m, respectively. When sesame oil was used, the reduction in surface tension was 42.19 ± 1.19 mN/m (mean of three measurements). For this oil, the CMD^−1^ and CMD^−2^ values were 52.39 ± 1.89 and 56.94 ± 3.96 mN/m, respectively. The worst results were obtained when pumpkin seed oil was used, as the mean value was 42.89 ± 1.16 mN/m, while the CMD^−1^ and CMD^−2^ values were 54.05 ± 1.78 and 59.9 ± 4.49 mN/m, respectively. The smallest differences between the CMD^−1^ and CMD^−2^ values were found for waste rapeseed oil and sunflower oil.

Statistically significant differences were found between the control group and the following oils: fresh rapeseed oil (*p* = 0.003), pumpkin seed oil (*p* = 0.002) and sesame oil (*p* = 0.004). In general, the highest average reduction in surface tension due to cell-free supernatant was observed for all oils after 120 h (40.68 ± 1.12 mN/m) and the lowest after 168 h (42.51 ± 0.99 mN/m).

### 2.2. Determination of Biomass

**Figure 1:**
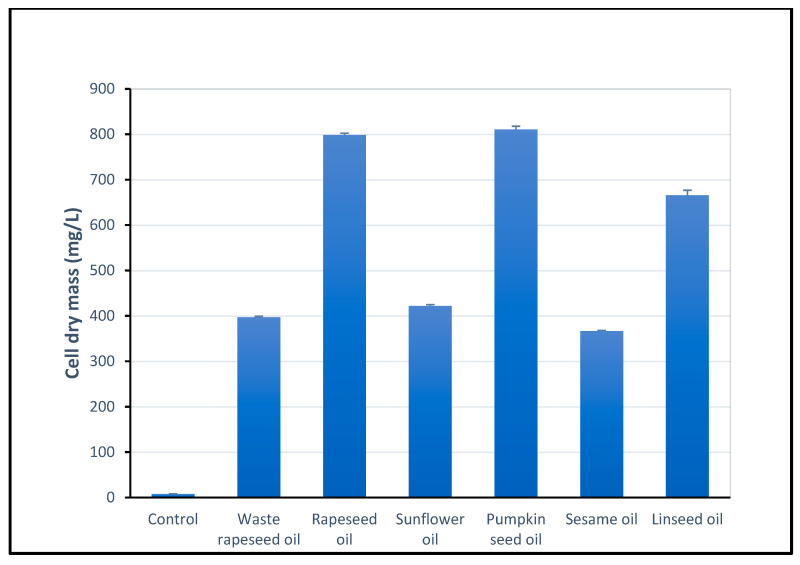
Cell dry mass (mg/L) obtained after 168 h of *S. marcescens* G8-1 cultivation with addition of different vegetable oils (n = 3).

The biomass obtained from the various vegetable oils is shown in Figure 1. The highest cell dry mass (CDM; 810 mg/L) was obtained by adding pumpkin seed oil. The biomass was similarly high when fresh rapeseed oil was used (798 mg/L CDM). A relatively high biomass concentration was achieved with linseed oil (665 mg/L CDM). The growth of *S. marcescens* was significantly lower when sunflower oil, waste rapeseed oil and sesame oil was added (422 mg/L, 397 mg/L and 366 mg/L CDM respectively).

### 2.3. Ultra-Performance Liquid Chromatography Coupled to Mass Spectrometry

**Figure 2:**
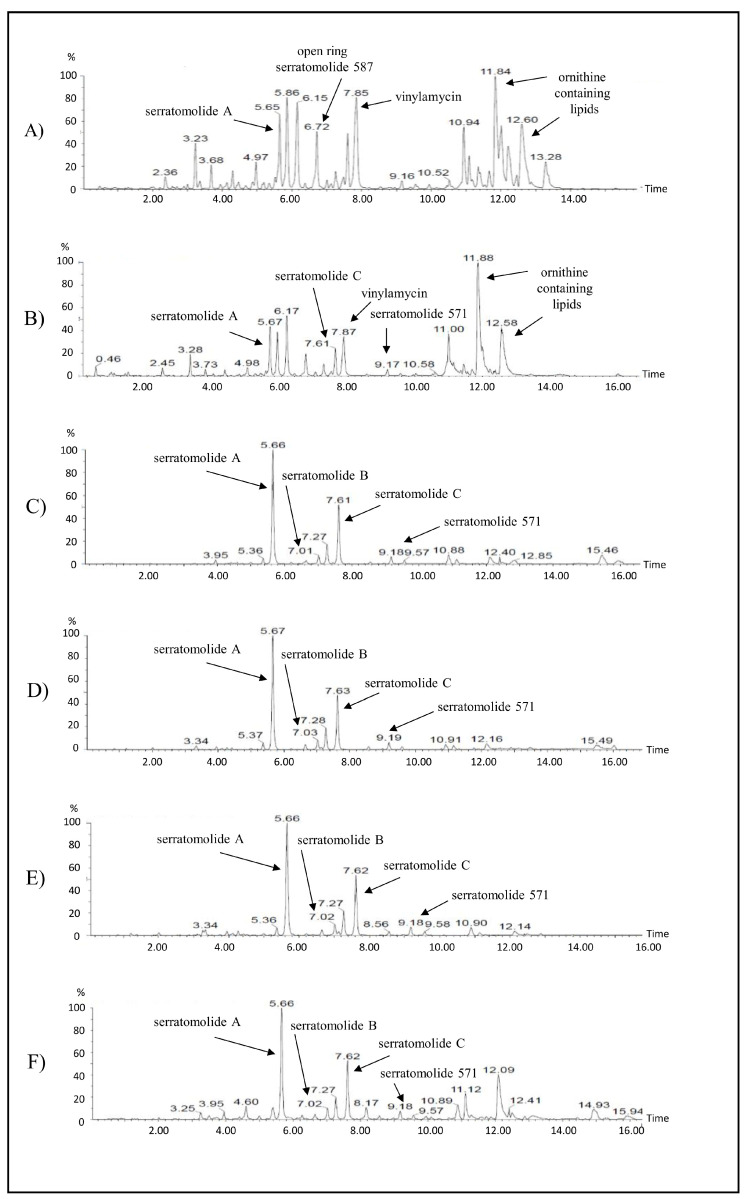
UPLC-ESI-MS analysis of compounds detected in cell-free medium supplemented with different vegetables oils. Serrawettin W1 (serratomolide A and its homologous), ornithine containing lipids, and vinylamycin are detailed. (**A**) waste rapeseed oil, (**B**) rapeseed oil, (**C**) sunflower oil, (**D**) pumpkin seed oil, (**E**) sesame oil, (**F**) linseed oil.

Ultra-performance liquid chromatography coupled to mass spectrometry UPLC-ESI-MS analysis revealed numerous peaks between 2 and 14 min (Table 2; Figure 2). The peaks corresponded to serratamolide A (Rt 5.65–5.67 min), serratamolide B (Rt 7.01–7.03 min), serratamolide C (Rt 7.61–7.62 min), serratamolide 571 (Rt 9.17–9.19 min) and the open ring serratomolide 587 (Rt 6.72 min). In addition, a peak was observed at a retention time of 11.84–11.88/12.58–12.60 min, which corresponded to ornithine-containing lipids. In addition, a peak corresponding to vinylamycin was observed at retention times 7.85 and 7.87. The opening ring serratomolide 587, ornithine-containing lipids and vinylamycin compounds was only observed in cultures with rapeseed oil (fresh and/or waste).

Furthermore, the addition of waste rapeseed oil induced the synthesis of serratomolide A and open ring serratomolide 587, whereas fresh rapeseed oil induced the synthesis of serratomolide A, C and 571. The addition of sunflower oil contributed to the synthesis of serratomolide A, B and C, while the addition of sesame or linseed oil contributed to the synthesis of serratomolide A, B, C and serratomolide 571. In case of pumpkin seed oil, synthesis of serratomolide A, B, and serratomolide 571 was observed.

### 2.4. Genomic Characterization

**Figure 3:**
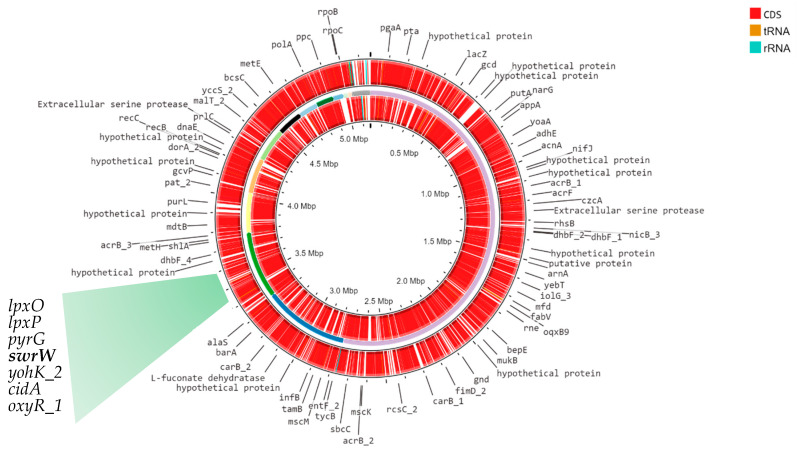
Genomic map of *S. marcescens* G8-1 created with PROKSEE software. The genes of NRSP cluster are detailed.

**Figure 4:**
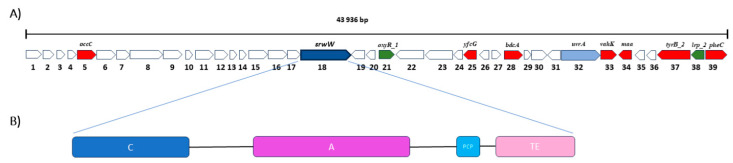
Genetic organization of NRSP cluster of serrawettin W1 constructed using antiSMACH platform. (**A**) core gene *srwW* (deep blue color), biosynthetic genes (red color), transport-related gene (blue color), and regulatory genes (green color) were identified. (**B**) gene coding for serrawettin consists of a condensation domain (C), an adenylation domain (A), a peptide carrier protein (PCP), and a thioesterase domain (TE).

The genomic sequence of *S. marcescens* G8_1 comprised 187 contigs with a total length of approximately 5,162,420 bp (Figure 3). There were 12 contigs longer than 10,000, while the longest contig was 2,766,183 bp. The GC content was 59.15%, while the number of predicted genes was 4465. The assembled genome was analyzed with antiSMASH to identify potential genes involved in serawettin synthesis. Analysis of the genome sequence led to the identification of a putative cyclic lipopeptide NRPS gene cluster (Figure 4). The total length of this cluster was 43,936 bp. Thirty-nine genes from the serrawettin W1 biosynthetic gene cluster were identified using the antiSMASH platform (Figure 4). These included eight biosynthetic genes, one transport-related gene and two regulatory genes. To confirm the finding of the cluster encoding serrawettin, the nucleotide sequence of NRPS was queried in NCBI BLAST and BLASTX (both BLAST+ 2.16.0). The result showed that the queried sequences were identical (100% identity) to the *srwW* gene of *Serratia marcescens* encoding serrawettin W1. AntiSMASH analysis showed that the *srwW* gene consists of a condensation domain, an adenylation domain, a peptide carrier protein and a thioesterase domain (Figure 4). PROKKA annotation showed that in addition to the *srwW* and *oxyR-1* genes, *lpxO*, *lpxP*, *pyrG*, *yahK* and *cidA* genes are also found in the NRPS gene cluster.

## 3. Discussion

*Serratia marcescens* G8-1 was selected as a microbial cell factory for the determination of the influence of plant oils on the synthesis of biosurfactants. The ability of the tested strain to produce biosurfactants was demonstrated in this study, as the cell-free medium was able to reduce the surface tension to 30.98 ± 0.46 mN/m without the addition of plant oils. The ability to reduce surface tension is a common screening method to detect the presence of biosurfactants produced by a microorganism [25]. Generally, a biosurfactant is considered effective if it can reduce the surface tension between water and air from 72 to 35 mN/m [26]. Since the strain *S. marcescens* G8-1 was even able to reduce the surface tension below 30 nM/m, this could indicate that this strain is a potential biosurfactant producer. The addition of vegetable oils had an effect on the production of biosurfactants, which was reflected in higher surface tension values (Table 1). Interestingly, the lowest surface tension value (38.83 ± 2.53 mN/m after 120 h) was found when waste rapeseed oil was used. Rapeseed oil contains a desirable fatty acid composition of predominantly unsaturated fatty acids and a healthy content of bioactive compounds [27]. Studies investigating the fatty acid composition of rapeseed oil indicate that the proportion of total unsaturated fatty acids (USFA) can reach almost 93.0%, but the polyunsaturated fatty acid content is rather low [28,29]. According to Panadare and Rathod [30], significant chemical processes such as oxidation, polymerization, hydrolysis and heat degradation take place during frying, which lead to changes in the content of saturated and unsaturated fatty acids. As a result of the hydrolysis process, vegetable oils with unsaturated and short-chain fatty acids are more easily absorbed than saturated and long-chain fatty acids due to their high water solubility. In addition, the formation of various chemical compounds may decrease or increase depending on the length of time the oil is fried, and the oil may also be contaminated with traces of metals from used equipment [31]. It is also likely that waste oil contains more nutrients such as fat, nitrogen, carbohydrates, protein, calcium, starch, magnesium and phosphorus, which are good for the growth of microorganisms, than refined oil [32]. Many authors have proven that the use of waste frying oil can lead to the synthesis of biosurfactants with higher efficiency [32]. An example of this is the work of Elkenawy and Gomaa [33], in which *S. marcescens* N2 used waste frying oil for the synthesis of biosurfactants. The results showed that the biosurfactant lowered the surface tension of water from 72 to 25.7 mN/m. However, in addition to waste oils, fresh vegetable oils are also frequently used for the production of lipopeptides by various *S. marcescens* strains. The results of Huang et al. [34] show that *S. marcescens* ZCF25 produces lipopeptides using olive oil. Cell-free supernatants reduce the surface tension from 72 to 26.59 mN/m. Soybean oil was also used as a carbon source for lipopeptide production with *S. marcescens* SmSA [35]. The results show that the surface tension can be reduced to 26 mN/m when this oil is used. For comparison, another agro-industrial substrate—wheat bran, was analysed by dos Santos et al. [36]. In this case, the cell-free supernatant of *S. marcescens* UCP 1549 reduced the surface tension to 28.4 mN/m.

The waste plant oils used as substrate could reduce the cost of biosurfactants and make this process even more environmentally friendly. Other oils which also had relatively low surface tensions are sunflower and linseed oils. Both contain a high concentration of unsaturated fatty acids, which can even reach more than 90% for sunflower oil [37] and almost 89% for linseed oil [38]. Sesame and pumpkin seed oils were less effective in reducing surface tension and contain less unsaturated fatty acids: 83.2 and 80.4%, respectively [28]. From this one could conclude that the concentration of unsaturated fatty acids correlates positively with the synthesis of biosurfactants, but only if the PUFA content is high. An exception is waste oils which, as a result of thermal conversion, have properties that promote the synthesis of biosurfactants. Unexpectedly, there was no correlation between the cell concentration in the culture medium and the efficiency of surface tension reduction (Figure 1 and Table 1). Taking both factors into account, the use of linseed oil is the most efficient, as the biomass was relatively high, as was the ability to reduce surface tension. However, these results must be interpreted with caution as they could be triggered by the fact that cultivation with different oils resulted in growth being at different stages. Accurate monitoring of bacterial growth was difficult as the emulsion was the result of oil addition.

The determination of the critical micelle dilution (CMD) of surfactants under different environmental conditions is important to determine the full properties of the surfactants studied. The detailed analysis of the dilutions of biosurfactants enables us to find the dilution point at which the micelles are no longer stable and begin to dissociate into individual surfactant monomers. As biosurfactants in many products are diluted during use, knowing the CMD helps to ensure that the biosurfactant remains effective after dilution. The CMD^−1^ and CMD^−2^ values obtained in this study are similar to those obtained by Ferraz, et al. [24] for vegetable oils. In our study, the addition of vegetable oils showed a limited effect on CMD values compared to the control group. In particular, similar CMD^−1^ values and even better CMD^−2^ values can be obtained with waste rapeseed oil and sunflower oil than in the control group. Since the dilution of the biosurfactant solution produced from waste rapeseed oil and sunflower oil only slightly changed the CMD values, we can assume that the potential of waste rapeseed oil and sunflower oil for the future production of lipopeptides using these two substrates is very promising

The cell-free medium of the strain *S. marcescens* G8-1 cultivated with plant oils was analysed by UPLC-ESI-MS. The major peaks detected in the cell-free supernatant correspond to serrawettin W1, in particular serratomolide A (m/z 515.3328 [M+H]+) and its analogues serratomolide B (m/z 541.3489), serratomolide C (m/z 543.3648) and serratomolide 571 (m/z 571.3958). In addition, open ring serratomolide 587 (m/z 587.3896) was also detected when waste rapeseed oil was used (Figure 2). These data are consisted with previous studies by Eckelmann, et al. [39] and Clements, et al. [40] who detected serratomolides in other strains of *S. marcescens*.

The antimicrobial activity of lipopeptides produced by *Serratia* species has been reported previously, although the mode of action is not yet fully understood [14]. Serrawettin W1 has been shown to have an inhibitory effect on a broad spectrum of Gram-positive bacteria, including various strains of *Staphylococcus aureus* (including methicillin-resistant *S. aureus*-MRSA) and *Staphylococcus epidermidis* [41]. In addition, Zhu et al. [18] indicated that serrawettin W1 produced by a *S. marcescens* S823 strain showed antifungal activity against *Candida albicans*. It was hypothesized that the broad-spectrum antimicrobial activity stems from the fact that the cell-free supernatant contains many homologs with two fatty acid chains of different lengths [14].

The peaks corresponding to ornithine-containing lipids were detected in the cell-free liquid from cultivation with rapeseed oils and to a lesser extent in cultivation with linseed oil (Figure 2). Ornithine lipids are acyl-oxyacyl lipids containing amino acids [42]. They are found in the membranes of many Gram-negative bacteria and some Gram-positive bacteria. The presence of ornithine lipids often appears to be part of a stress response to changing environmental conditions [43]. The amount of ornithine lipids in different cultivations was largely dependent on the current growth conditions. A compound with a molecular ion at m/z 494.3252 [M+H]+ was detected in the cell-free liquid with rapeseed oil. The elemental composition of the protonated molecular ion with m/z 494.3252 confirmed the molecular formula C_26_H_43_N_3_O_6_ which corresponds to vinylamycin. The structural study revealed that vinylamycin is a fourteen-ring depsipeptide consisting of a molecule of D-valine, L-alanine and 4-amino-penta-2,4-dienoic acid and 2-hydroxy fatty acid. To date, the synthesis of vinylamycin has only been demonstrated in Streptomyces sp. This substance has been shown to has antimicrobial activity against Gram-positive bacteria, including MRSA [44]. It could be hypothesized that the biocidal cocktail of *Serratia* consists not only of prodigiosin and biosurfactants [45], but also of vinylamycin. Recently, Wang, et al. [46] showed that vinylamycin is active against K562 leukemia cells. The discovery that *S. marcescens* can produce vinylamycin may indicate that the synthesis of this substance may be more widespread in the bacterial world. In addition, the data presented here describe the first assumptions on vinylamycin production by *S. marcescens*, which can be used to improve the efficiency of synthesis of this substance and to test its biological activities.

The molecular ability to synthesize serrawettin W1 was confirmed by genomic analysis. The result showed that the DNA sequences of the investigated strain G8-1 were identical (100% identity) to the *srwW* gene of *S. marcescens*, which codes for serrawettin W1. Using the AntiSMASH platform, it was showed that the *swrW* gene consists of a condensation domain, an adenylation domain, a peptide carrier protein and a thioesterase domain (Figure 4). This organization is typical of *S. marcescens* [2,47]. AntiSMASh found eight central biosynthetic genes, one transport-related gene and two regulatory genes. Information on regulatory genes of lipopeptide synthesis is scarce. The regulatory genes identified by antiSMASh are *OxyR1*, which is located in the immediate vicinity of *srwW*, and the more distant *lrp_2* gene. The *OxyR* gene is one of the transcriptional regulators of the LysR family, which is conserved in many Gram-negative and Gram-positive bacteria. The results of Shanks, et al. [48] showed that the *oxyR* gene is required for biofilm formation in *S. marcescens*, leading the authors to model that *oxyR* contributes to biofilm formation in *S. marcescens* as a positive regulator of fimbrial expression. Here, we propose that *oxyR* is responsible for *swrW* regulation, which drives the process of serratomolide synthesis. This could be supported by the results of Marques-Pereira, et al. [47], who also found a gene encoding the *LysR* regulatory protein in a serratomolide W1 biosynthetic gene cluster. The second regulatory gene (*lrp_2*) is a leucine-responsive transcriptional regulator that controls the transcription of several operons, including those for amino acid synthesis and degradation, nutrient transport and pili production [49]. Hay, et al. [50] found that *Paracoccus mirabilis*, which lacks Lrp, does not engage in flagellation and swarming activity. A similar observation was made when studying *Escherichia coli* [51]. This may support our hypothesis that the *lrp_2* gene is also involved in the process of regulating serrawettin synthesis.

## 4. Materials and Methods

### 4.1. Strain

*Serratia marcescens* sp. G8_1 was isolated from activated sludge [52]. This strain shows ability to produce biosurfactants and red pigment prodigiosin.

### 4.2. Cultivation

The inoculum was prepared using Luria Bertani medium (tryptone 10 g/L, yeast extract 5 g/L, NaCl 10 g/L) with addition of glucose 10g/L. Bacteria were cultivated on medium containing 0.5% of yeast extract supplemented with 3% different plant oils at pH 7.0. The following plant oils were used: rapeseed oil, linseed oil, pumpkin seed oil, sunflower oil, and sesame oil. Waste rapeseed oil, which was previously used for frying food, was additionally included to experiment. Erlenmayer flasks containing 100 mL of medium were incubated in orbital shaker (150 rpm) at 30 °C for 168 h. After incubation, the broth culture was centrifuged at 10,000 rpm for 20 min at 4 °C to obtain the cell free supernatant. Biomass was lyophilized and dry biomass was determined. Each cultivation was performed in triplicate.

### 4.3. Surface Tension Measurements

The surface tension of the cell free supernatant was measured using a Krüss K 100 processor tensiometer (KRÜSS GmbH, Hamburg, Germany), using the Wilhelmy plate method. The bacterial cells were harvested by centrifuging the culture at 5500 rpm for 20 min (MPW-351 centrifuge, MPW Med. Instruments, Warszawa, Poland). The surface tension of the biosurfactant compounds present in the cell free supernatant was measured at room temperature. Prior to surface tension measurements of each cell free supernatant sample, calibration was performed using distilled water to ensure validity of the measurements. Each biological replicate was measured in triplicate and an average value was recorded as the surface tension of the sample. Critical micelle dilution (CMD), a parameter used as an indirect measure of surfactant concentration, was determined by measuring the surface tension of serial dilutions of the cell-free broth in distilled water at pH 7.0 [24]. Milli-Q water with surface tension of 72 mN/m was used to calibrate the tensiometer. The significance of the differences between the values of the surface tension of the individual oils and the control group was tested using the Kruskal-Wallis test and the subsequent Dunn’s post-hoc test.

### 4.4. Ultra-Performance Liquid Chromatography Coupled to Mass Spectrometry

The compounds present in the cell-free supernatant were analysed by ACQUITY UPLC I-Class chromatography system (Waters Corporation, Milford, MA, USA) coupled with SYNAPT G2-Si mass spectrometer equipped with an electrospray ion source and quadrupole-Time-of-Flight mass analyser (Waters Corp., Milford, MA, USA). For the chromatographic separation of analytes an Acquity UPLC^®^ HSS T3, 1.8 μm particle size, 2.1 × 100 mm (Waters Corporation) thermostated at 40 °C temperature was used. Mobile phases consisted of 0.1% formic acid (solvent A) and 0.1% formic acid in acetonitrile (solvent B). A gradient program was developed as follows 45% B (0–0.5 min), 45–95% B (0.5–13.0 min), 95–95% B (13.0–14.0 min), 95–45% B (14.0–14.1 min) and 45–45% B (14.1–17.0 min). The injection volume was 3 μL and the flow rate was 0.3 mL/min. For mass spectrometric detection the electrospray source was operated in a positive, high resolution mode. To ensure accurate mass measurements, data were collected in centroid mode. The mass was corrected using leucine enkephalin solution as an external reference (Lock-SprayTM, Chattanooga, TN, USA). The optimized source parameters were: capillary voltage 2.9 kV, cone voltage 20 V, source temperature 120 °C, desolvation gas (nitrogen) flow rate 650 L/h with the temperature 285 °C, nebulizer gas pressure 6.5 bar. Mass spectra will be recorded over an m/z range of 100 to 2000. The data obtained was analysed using the Masslynx software version 4.1 (Waters Corporation).

### 4.5. Genomic Characterization

Genomic DNA was extracted from this overnight culture using a Genomic Mini (A&A Biotechnology, Gdańsk, Poland) according to the manufacturer’s recommendation. The result of extraction was analysed using QUBIT 2.0 whereas DNA integrity was checked by gel electrophoresis on a 0.8% Agarose gel. Whole-genome shotgun sequencing was performed by Illumina MiSeq (paired end, 2 × 300 bp) with DNA libraries prepared using the TruSeqHT (Illumina, San Diego, CA, USA) according to the manufacturer’s recommendation. Trimmomatic v 0.32 (2) was used to remove barcodes and trim reads with a window size of 4 and a Q score cutoff of 20. Quality analyses of reads were performed with FastQC v 0.11.9 (https://www.bioinformatics.babraham.ac.uk/projects/fastqc/, accessed on 15 September 2024).

Trimmed reads were uploaded to KBase platform [53] for further analysis. De novo genome assembly was performed using SPAdes v. 3.15.0 [54], and genome assembly metrics were calculated with QUAST 5.0.2. [55]. Serrawettin biosynthetic gene cluster prediction was performed using the web platform antiSMASH 3.0 [56]. BLAST (BLAST+ 2.16.0) analysis was performed for *swrW* gene both on nucleotides and proteins. The PROKSEE (v. 1.7) server was used to annotate genome [57] and generate high-quality navigable maps of genome. The sequencing data was deposited at the National Center for Biotechnology Information (NCBI) Genebank database with accession number PRJNA1218503.

## 5. Conclusions

The newly isolated strain of *S. marcescens* G8-1 shows the ability to produce serraweratin, apart from the production of prodigiosin. Waste rapeseed oil showed the best results in reducing surface tension. This oil also showed the smallest differences between CMD^−1^ and CMD^−2^ values. The present results indicate that the cultivation period of 168 h is too long and should be shortened to at least 120 h. The investigated strain has the ability to produce serrawettin W1, which was confirmed by genome analysis. The genome annotation suggests that genes from the LysR family (*oxyR_1*) and the leucine-responsive transcriptional regulator (*lrp_2*) may be involved in the process of *swrW* regulation. Serratomolide A and its homologs were determined in cell-free culture medium. Interestingly, it was shown for the first time that *S. marcescens* can synthesize vinylamycin—a substance with antimicrobial and anticancer activity.

## Figures and Tables

**Table 1:** Reduction of the surface tension of the cell-free growth medium for *Serratia marcescens* G8-1 supplemented with various vegetable oils (n = 3).

	Surface Tension (mN/m) After 120 h	Surface Tension (mN/m) After 144 h	Surface Tension (mN/m) After 168 h	Surface Tension (mN/m) Mean (for Three Measurements)	CMD^−1^ (mN/M)	CMD^−2^ (mN/M)
Oil free	29.84 ± 0.45	31.28 ± 0.18	30.985 ± 0.46	30.7 ± 0.76	48.41 ± 1.69	58.135 ± 2.22
Fresh rapeseed oil	42.02 ± 3.39	40.67 ± 1.06	43.19 ± 4.44	41.96 ± 1.26	52.6 ± 3.33	56.12 ± 1.49
Waste rapeseed oil	38.83 ± 2.53	40.87 ± 0.76	41.45 ± 0.45	40.38 ± 1.37	49.94 ± 1.13	52.16 ± 1.38
Linseed oil	40.5 ± 0.81	41.1 ± 1.07	41.78 ± 1.15	41.12 ± 0.64	50.53 ± 2.05	55.1 ± 3.32
Pumpkin seed oil	41.64 ± 0.34	43.1 ± 1.67	43.94 ± 1.10	42.89 ± 1.16	54.05 ± 1.78	59.9 ± 4.49
Sunflower oil	40.31 ± 1.30	42.03 ± 0.42	41.73 ± 0.39	41.35 ± 0.92	50.09 ± 2.32	53.11 ± 2.92
Sesame oil	40.81 ± 2.12	42.79 ± 2.00	42.97 ± 1.75	42.19 ± 1.19	52.39 ± 1.89	56.94 ± 3.96

**Table 2:** Summary of the compounds identified in cell-free growth medium for *Serratia marcescens* G8-1, detected using high-resolution mass spectrometry (UPLC-ESI-MS).

Compound Name	UPLC Rt	Molecular Formula	m/z [M+H]+	m/z [M+H]+	Oil
				Theoretical	
Serratomolide A	5.65	C_26_H_46_N_2_O_8_	515.3328	515.3332	rapeseed waste
	5.66				sunflower; sesame; linseed
	5.67				rapeseed, pumpkin
Serratomolide B	7.01	C_28_H_48_N_2_O_8_	541.3489	541.3489	sunflower
	7.02				sesame; linseed
	7.03				pumpkin
Serratomolide C	7.61	C_28_H_50_N_2_O_8_	543.3648	543.3645	rapeseed; sunflower
	7.62				linseed
Serratomolide 571	9.17	C_30_H_54_N_2_O_8_	571.3958	571.3958	rapeseed
	9.18				sesame; linseed
	9.19				pumpkin
Open ring serratomolide 587	6.72	C_30_H_54_N_2_O_9_	587.3896	587.3908	rapeseed waste
Ornithine containig lipids	11.84	C_37_H_72_N_2_O_5_	625.5518	625.5505	rapeseed waste
	11.88				rapeseed
	12.58	C_40_H_76_N_2_O_5_	665.5831	665.5832	rapeseed
	12.60				rapeseed waste
Vinylamicine	7.85	C_26_H_43_N_3_O_6_	494.3252	494.3230	rapeseed waste
	7.87				rapeseed

## Data Availability

The sequencing data was deposited at the National Center for Biotech nology Information (NCBI) Genebank database with accession number PRJNA1218503.
